# Food insecurity and men’s perpetration of partner violence in a longitudinal cohort in South Africa

**DOI:** 10.1136/bmjnph-2021-000288

**Published:** 2022-02-07

**Authors:** Abigail M Hatcher, Torsten B Neilands, Dumisani Rebombo, Sheri D Weiser, Nicola J Christofides

**Affiliations:** 1 Department of Health Behavior, Gillings School of Global Public Health, University of North Carolina System, Chapel Hill, North Carolina, USA; 2 School of Public Health, Faculty of Health Sciences, University of the Witwatersrand, Johannesburg, South Africa; 3 Department of Medicine, University of California San Francisco, San Francisco, California, USA; 4 Sonke Gender Justice, Johannesburg, South Africa; 5 School of Public Health, University of the Witwatersrand Faculty of Health Sciences, Johannesburg, Gauteng, South Africa

**Keywords:** malnutrition, mental health, preventive counselling

## Abstract

**Background:**

Although food insecurity has been associated with intimate partner violence (IPV), few studies examine it longitudinally or among male perpetrators.

**Methods:**

We used secondary data from a trial that followed 2479 men in a peri-urban settlement in South Africa (February 2016–August 2018). Men self-completed questionnaires at baseline (T0), 12 months (T1) and 24 months (T2) on food security, household type, relationship status, childhood abuse exposure, alcohol use, and perpetration of physical and/or sexual IPV. Cross-lagged dynamic panel modelling examines the strength and direction of associations over time.

**Results:**

At baseline, rates of IPV perpetration (52.0%) and food insecurity (65.5%) were high. Food insecure men had significantly higher odds of IPV perpetration at T0, T1 and T2 (ORs of 1.9, 1.4 and 1.4, respectively). In longitudinal models, food insecurity predicted men’s IPV perpetration 1 year later. The model had excellent fit after controlling for housing, relationship status, age, childhood abuse and potential effect of IPV on later food insecurity (standardised coefficient=0.09, p=0.031. root mean squared error of approximation=0.016, comparative fit index=0.994). IPV perpetration did not predict later food security (p=0.276).

**Conclusion:**

Food insecurity had an independent, longitudinal association with men’s IPV perpetration in a peri-urban South African settlement. These findings suggest food security could be a modifiable risk factor of partner violence.

**Trial registration number:**

NCT02823288.

**What this paper adds**

- This paper is the first to examine food insecurity and intimate partner violence (IPV) perpetration longitudinally.
- Among 2384 men living in a peri-urban South African settlement, rates of past-year food insecurity (65.5%) and IPV perpetration (52.0%) and were high.
- We found a small but significant effect of men's self-reported food insecurity on their use of violence a year later.

## Introduction

Intimate partner violence (IPV) is a major burden to human rights and health across the globe, with one-quarter of women reporting IPV exposure in their lifetime.^1^ Despite considerable research to understand the predictors of women’s IPV exposure, less is known about the factors associated with men’s IPV perpetration.

Poverty may be a strong underlying driver of men’s IPV perpetration. North American cross-sectional research shows that men who are unemployed have higher rates of perpetrating IPV,^2 3^ as do men with lower income.^4^ Cross-sectional research from India finds that men with fewer household assets have greater odds of perpetrating IPV.^5 6^ An eight-country study across sub-Saharan Africa identified no relationship between poverty and IPV perpetration.^7^ In rural South Africa, men who were better off (in contexts of high poverty) were *more* likely to enact violence against a partner.^8^

This complex cross-sectional relationship is only starting to be parsed out in longitudinal research. A recent systematic review identified fewer than a dozen longitudinal studies on IPV perpetration, and all were conducted in high-resource settings.^9^ Two longitudinal studies of poverty and men’s IPV perpetration offer mixed evidence. Krishnan and colleagues learnt in India that men whose employment opportunities worsened had higher odds of perpetrate IPV.^10^ Fox and colleagues found in the USA, however, that while employment status had no association with later IPV, financial well-being at baseline was strongly associated with IPV perpetration 6 years later.^11^

Preliminary evidence suggests a relationship between food insecurity—a sensitive marker of poverty—and IPV. When measured using a validated scale, food security scales provide a ‘snapshot’ of a household situation with regards to meeting basic needs.^12^ Food insecurity is associated with increased odds of IPV exposure among women^13–15^ and increased odds of men’s perpetration of IPV.^16 17^ In a systematic review of the literature, all extant literature on men’s perpetration of IPV and food insecurity is cross-sectional,^18^ highlighting the need for longitudinal research on these intersecting conditions.

One conceptual challenge to assessing cross-sectional research on poverty and IPV perpetration is that violence and poverty may have a bidirectional relationship. Evidence among women survivors shown that IPV has a negative effect on economic earning potential years after the violence occurs.^19^ Less is known about IPV leading to poverty among male perpetrators, but there are several plausible explanations for a link. IPV perpetration may lead to greater household poverty if it increases financial burden for the family. This is consistent with research that demonstrates a high cost associated with the injuries, mental debilitation and loss of work for IPV survivors.^20^ Alternately, IPV perpetration could make relationships more unstable, leading to a decreased ability of the household to secure food. Finally, IPV perpetration is strongly associated with unplanned pregnancy,^21^ a life event that could worsen household poverty as resources are extended.^22^

## Methods

The current study aims to determine the direction and strength of the longitudinal association between food insecurity and men’s perpetration of IPV. Secondary data from a cluster randomised control trial were collected from men at three-time points in a peri-urban setting in South Africa.

### Data collection

Trained research assistants recruited a volunteer sample of men from a peri-urban settlement (also called a former ‘township’) near Johannesburg. The settlement has high levels of poverty and residents perceive safety to be very low.^23^ While some houses are brick, electrified and have running water, many are shacks constructed with sheet metal who use community taps and toilets.

The research was conducted during the period of February 2016–August 2018. Eligible men lived in a predefined research area (called a cluster) for at least 12 months and were aged 18–40 years. The sample was recruited by a local mobilisation team who used convenience sampling methods during daytime hours at local places (schools, street corners, outside restaurants) within a total of 18 clusters for a cluster randomised controlled trial.

After taking part in the baseline (T0) questionnaire, a community even was held to randomly allocated clusters to an intervention arm or a control arm of the trial. The intervention has been detailed elsewhere,^24^ but briefly it involved community mobilisation around the issue of IPV through group meetings, door-to-door communication campaigns and engaging local leaders. Men participating in the trial lived in areas of intervention or control work, but may not have directly participated. These men were followed up approximately 12 months after baseline (T1) and again at 24 months after baseline (T2).

Research assistants asked participants at baseline (T0) to complete a locator form with contact names and numbers of the participant and close friends or family. Participants were contacted via phone call and text message at T1 and T2 and invited to complete the questionnaire at a convenient place in their neighbourhood. Efforts to trace men consisted of: multiple calls to cell phones, contacting next of kin, friends and other participants who had listed them as friends, home visits to addresses where men reported living, walks around the cluster to ask neighbours, and home visits to other provinces, cities and neighbourhoods within Johannesburg.

Data collection was conducted in the language of participant choice (English, isiZulu, Tswana or Sepedi) on tablet computers using audio-computer assisted data collection (ACASI) software. ACASI allows important data to be collected about socially desirable and undesirable (including illegal) activity while ensuring anonymity. Data from tablets were uploaded multiple times daily to an encrypted server housed at the University of the Witwatersrand.

Participation was on the basis of written, informed consent and each participant was reimbursed R50 (approximately US$3.50) at T0, R100 (US$7) at T1 and R150 (US$10.50) at T2. These participant reimbursement rates were somewhat lower than those used by South African clinical trials because participants did not need to travel far—each study visit was walkable within about 10 min by foot. We anticipate this lower rate helped reduce the economic incentives of participation, though in a setting where work is scarce it is plausible that some men took part due to this reimbursement.

Researchers received intensive training on IPV, the study protocol, collecting sensitive information, and ensuring data quality and participant confidentiality. Study procedures complied with ethical recommendations of the United Nations Multi-Country Study on Men and Violence.

Community members were involved before quantitative research started, through a series of in-depth interviews with men living within the area, meetings with civil society organisations, and a community advisory board. These formative steps informed the development of research questions and the design of the trial questionnaire. Local interviewers were hired to conduct the research and lead recruitment of community members into the study. We held three community workshops: prior to trial start a new study with men about relationships was introduced without using the term ‘violence’; on baseline data collection completion we randomly selected which neighbourhoods would be in intervention or control arms of the trial; after the trial was analysed we shared information about the null primary findings (ie, the intervention had no effect on men’s IPV perpetration).

### Measures

#### Dependent variable

*IPV perpetration* was measured as an index of items on physical and sexual violence towards a current or ex-partner in the past year using the WHO Multi-Country Study Instrument^25^ (a full version of which can be found here^26^). A total of 17 items asked about behaviourally specific acts (eg, hitting, choking, forcing sex) with answers on a Likert-type scale scored as 0 (never), 1 (once), 2 (two to three times), 3 (four or more times). IPV was defined in two ways. In logistic regression it was defined dichotomously. A person who reported using one or more forms of psychological, physical and/or sexual violence was considered as having past-year IPV perpetration. In the dynamic panel modelling IPV was treated as a continuous measure of intensity (summing the responses with a range of 0–39). Internal consistency of the IPV instrument was strong (Cronbach’s α=0.93, 0.93, 0.94 at baseline, midline and endline, respectively). In supplemental analysis IPV was defined as a dichotomous measure (any IPV perpetration vs none).

#### Time-variant explanatory variables

*Food insecurity* is defined as having uncertain or limited availability of nutritionally adequate food or the inability to acquire safe, acceptable foods.^27^ Beyond sheer hunger from insufficient food intake, food insecurity also includes poor dietary quality and worry or anxiety over securing food supplies.^28^ Food insecurity was measured using three items of the Household Food Insecurity Access Scale that have been validated as a measure of household hunger^29^: (1) having no food in the house, (2) going to sleep hungry and (3) going without food.^29^ In addition, two items important to this highly impoverished setting were added (4) borrowing food because there was not enough, and (5) stealing food because there was not enough. Each item has a Likert-type response ranging from never (=0), rarely (=1), sometimes (=2) or often (=3). Food insecurity was assessed as a continuous measure of a total scale score (range 0–15). Internal consistency at all time points was acceptable (Cronbach’s α=0.84, 0.87, 0.86 at baseline, midline and endline, respectively). In supplemental analysis, food insecurity was defined as a dichotomous outcome (food secure vs food insecure) using validated cutoffs.^29^

*Housing* was assessed using a single (non-validated) item developed during our formative research that asks about what home a person lives in. The options are ranked by order of more affluent (ie, owning one’s home or living in a government-funded house) to more impoverished (ie, living in a shack behind another house, living in a single outside room). Housing was operationalised as a quasi-continuous variable (range 1–6), with a higher score indicating greater housing insecurity. *Relationship status* was assessed through a single item asking a participant whether he was married and living together, non-married but living together, married and living apart, non-married but living apart or single. This was used dichotomously (single vs not) and as a quasi-continuous variable (range 1–5) with higher scores indicating more relational distance. *Employment* was asked through a single Likert-type item asking how often they worked in the past 12 months: never (=0), rarely (=1), sometimes (=2) or often (=3).

#### Time-invariant explanatory variables

We controlled for baseline *age* in years as a sociodemographic variable as men tend to ‘age out’ of IPV perpetration as they get older.^30^ *Intervention exposure* was a dichotomous covariate depending on whether the participant was randomly assigned to an intervention cluster or not. Since this had no measurable effect on food insecurity or IPV exposure it was omitted from the final models.

We controlled for baseline reports of *childhood abuse* since this predictor strongly influences men’s adult use of IPV.^16^ *Childhood abuse* was measured using 15-item revised Childhood Trauma Questionnaire, a shortened version of an instrument that has been used previously in South Africa. The tool asked participants to self-report frequency of emotional, physical and sexual abuse before the age of 18 (4 items, 2 items, and 3 items, respectively), and whether they witnessed their mother being beaten by her husband or boyfriend. A higher score reflected more severe levels of childhood trauma and the internal consistency was good (Cronbach’s α=0.85).

### Analysis

This secondary data analysis was not pre-registered. We first used descriptive statistics to describe the cohort at baseline measurement using svy commands in StataMP V.16 (StataCorps). Bivariate inferential statistics accounting for the clustered nature of the data estimate the cross-sectional association between IPV perpetration and our main variables of interest.

The first models we assessed were cross-sectional and lagged logistic regressions adjusting for clustering by neighbourhood using svy commands. The purpose in estimating these models was to identify the magnitude and direction of the relationship between food insecurity and IPV perpetration, without regard to reciprocal effects.^31^ These also allowed for ease of interpretation since they report ORs.

We then estimated cross-lagged dynamic panel data models (DPMs). Described fully elsewhere,^32^ cross-lagged DPMs are a relatively new analytic technique that unite cross-lagged and fixed effects in the same model using a structural equation modelling (SEM) framework. Fixed effects are useful as they eliminate the effects of all time-invariant confounders even when the potential confounders are unmeasured or undefined.^31^ Fixed effects models estimate within-person changes,^33^ in this case determining how a person’s food security level changes over time and how this is related to their IPV perpetration.

We estimated DPMs that incorporated reciprocal, lagged effects of the key variables: food insecurity and IPV perpetration.^31^ Unlike traditional fixed effects-only models, creating DPMs within the SEM framework allows estimation of two or more variables that may have lagged, reciprocal effects on each other.^34^ This is important since it is plausible that IPV perpetration could lead to future food insecurity. The lagged, reciprocal effects of IPV perpetration on future food insecurity are included in the SEM model by regressing food security in T2 against IPV perpetration and other covariates measured in T0 and T1.^32^

In addition to food insecurity, we extended the unadjusted models described above to include housing status and relationship status. As employment status alcohol use did not demonstrate statistical significance at the p<0.20 value, we removed them from the final model. In conducting DPMs within the SEM framework, one has the option of considering how past violence use influences later violence use. We incorporated this into the model by controlling for IPV perpetration lagged by one timepoint.

All lags were 1 year, as data collection occurred at baseline, 12 months, and 24 months. We use maximum likelihood with missing values to address missing values. Final model estimates are presented after adjusting for clustering by neighbourhood.

## Results

The cohort was composed of 2479 men at T0, of whom 1508 (63%) were followed to T2, 24 months after study enrolment (table 1). Men were a median of 27 years old (range: 18–40 years) at T0.

**Table 1:** Descriptive statistics at three study timepoints

	T0	T1	T2
	(n=2384)	(n=618)	(n=1508)
	Median (IQR) or number (%)	Median (IQR) or number (%)	Median (IQR) or number (%)
**Dependent variable**			
IPV perpetration	1288 (52.0%)	291 (47.1%)	542 (34.9%)
**Time variant variables**			
Food insecure	1530 (65.5%)	307 (51.3%)	838 (55.9%)
Relationship status: single	391 (16.4%)	109 (17.6%)	176 (11.7%)
Lives in shack or single room	1466 (62.0%)	364 (59.1%)	959 (65.0%)
Never worked in past-year	873 (36.5%)	74 (12.1%)	262 (17.5%)
Problem drinking	914 (39.0%)	203 (33.7%)	470 (31.8%)
**Time invariant variables**			
Age	27 (23–32)	27 (24–32)	28 (25–34)
Any childhood abuse	1962 (82.2%)	–	–

All time variant variables and the dependent variables are presented as at baseline.

IPV, intimate partner violence.

A total of 1288 (52.0%) men reported perpetrating physical and/or sexual IPV in the past year at T0. This proportion was consistent at T1, when 47.1% enacting past-year IPV. At T2 542 men (34.9%) reported past-year IPV.

**Figure 1:**
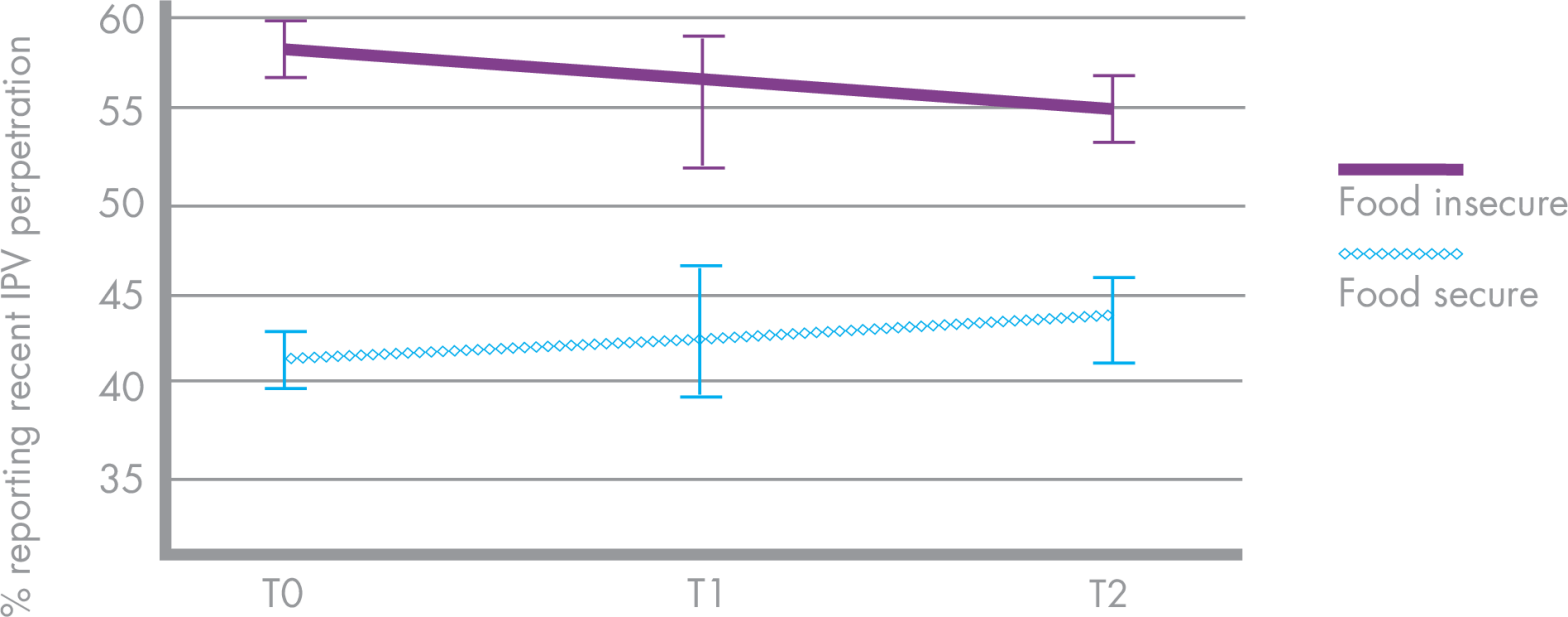
Men’s intimate partner violence (IPV) perpetration by food security status.

Food insecurity was consistently reported over time. At T0, 65.5% participants reported food insecurity. At T1 the proportion was similar (51.3%) and by T2 it had increased slightly (55.9%, respectively). Men who were food insecure at baseline reported considerably higher rates of IPV at all three timepoints (figure 1).

### Bivariate analysis

In clustered bivariate analysis, food insecurity was associated with higher IPV perpetration at multiple timepoints (table 2). Men reporting food insecurity at T0 had higher odds of past-year IPV use at T0 (OR=1.85, 95% CI 1.61 to 2.12), at T1 (OR=1.40, 95% CI 1.01 to 1.96) and at T2 (OR=1.36, 95% CI 1.36 to 1.69). Not all associations reached statistical significance but all show a similar direction.

**Table 2:** Association between food insecurity and men’s perpetration defined as physical and/or sexual IPV

	Any IPV perpetration		
	T0	T1	T2
	OR	OR	OR
Food insecure T0	1.85***	1.40^*^	1.36**
Food insecure T1	1.02	1.31	1.05
Food insecure T2	1.17	1.35	1.31**

Logistic regression models account for clustering by neighbourhood.

*p<0.05, **p<0.01, ***p<0.001

IPV, intimate partner violence.

### Cross-lagged dynamic panel models

We present cross-lagged dynamic panel models that lag food insecurity by 1 year and model the effects on later IPV perpetration (table 3). In model 1, only food insecurity is considered and changes in food are associated with a small but significant increase in IPV perpetration 1 year later. Every standardised increase in food insecurity is associated with a 0.07 (p=0.045) SD increase in IPV intensity. Model fit indices are good (root mean squared error of approximation (RMSEA)=0.044, comparative fit index (CFI)=0.984).

**Table 3:** Cross-lagged dynamic panel models examining intensity of IPV perpetration (n=2479)

	Model 1			Model 2*		
	Coef	SE	P value	Coef	SE	P value
**Time variant variables**						
Food insecurity	0.07	0.04	0.045	0.09	0.04	0.031
Housing status	–	–	–	0.12	0.05	0.009
Relationship status	–	–	–	0.12	0.04	0.153
**Time invariant variables**						
Age at baseline	–	–	–	−0.10	0.03	<0.001
Childhood abuse	–	–	–	0.21	0.03	<0.001
Alpha	0.77	0.05	<0.001	0.59	0.06	<0.001
**Fit indices**						
Chi^2^	6.56			7.64		
Chi^2^ p value	0.010			0.106		
Df	1	**Lower bound**	**Upper bound**	4	**Lower bound**	**Upper bound**
RMSEA	0.044	0.015	0.082	0.016	0.000	0.035
CFI	0.984			0.994		

Models account for past use of violence and bidirectional nature of association (ie, IPV perpetration leading to later food insecurity).

CFI, comparative fit index; Coef, standardised coefficient; IPV, intimate partner violence; RMSEA, root mean squared error of approximation.

**Figure 2:**
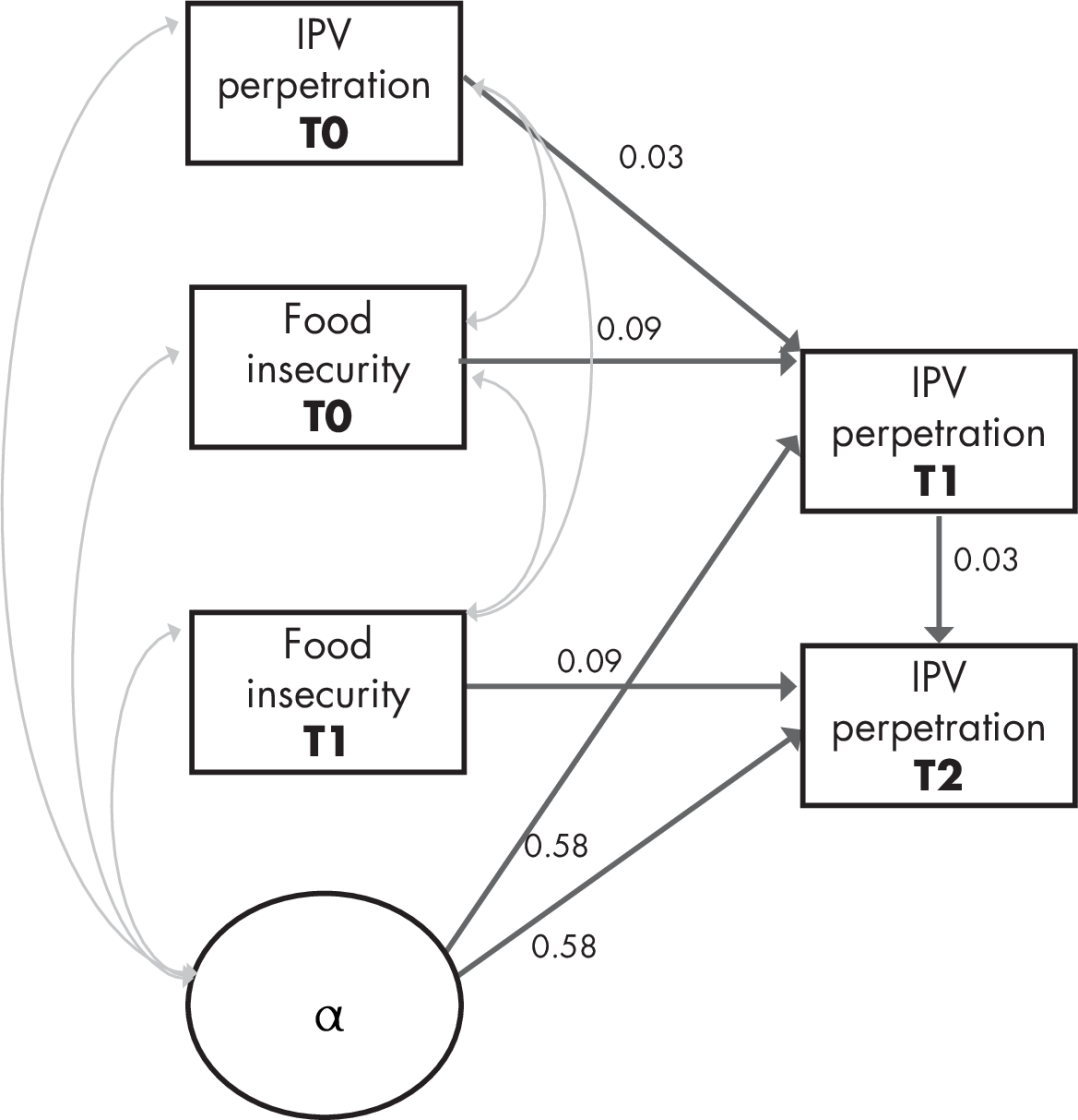
Path diagram for three-wave dynamic panel model of food insecurity to later IPV perpetration (adapted with permission from Allison *et al* 32). All p<0.05, estimates are standardised coefficient, root mean squared error of approximation: 0.016 (0.001 to 0.035), comparative fit index: 0.994. Model accounts for clustering by neighbourhood, age, housing, relationship status, childhood exposure to abuse and bidirectionality of IPV towards later hunger. IPV, intimate partner violence.

In model 2, we added a time-invariant measure (childhood abuse exposure) and time-variant predictors (housing status, relationship status and past IPV perpetration). In this model, food insecurity retains its consistent association with later IPV perpetration (coef=0.09, p=0.034) even after controlling for other covariates and bidirectionality of IPV leading to later food insecurity. Declines in housing status have a similar magnitude of effect on later IPV perpetration (coef=0.12, p=0.009) as does younger age (coef=−0.10, p<0.001). Childhood abuse exposure has a strong effect on IPV perpetration (coef=0.24, p<0.001). Fit indices are excellent (RMSEA=0.016, CFI=0.994, figure 2).

We examined lagged IPV use as a predictor of later food insecurity, while controlling for housing, age at baseline and childhood abuse (online supplemental table 1). In this model, men’s IPV perpetration is not a driver of later food security (p=0.276). On the other hand, age at baseline and childhood abuse do predict adult food insecurity (p<0.001 and p<0.001, respectively). Model fit was poor (RMSEA=0.080, CFI=0.661), suggesting that a reverse causal hypothesis of IPV perpetration leading to later food insecurity do not fit these data.

10.1136/bmjnph-2021-000288.supp1 Supplementary data

## Discussion

In this study, we found that food insecurity had significant association with men’s IPV perpetration 1 year later. Of 2479 men from South Africa, roughly half (52.0%) reported recent use of IPV and a majority (65.5%) were food insecure at baseline. Food security was associated with higher cross-sectional odds of IPV perpetration. Men’s IPV perpetration was predicted by their food security 1 year prior. The measurable longitudinal effect of food security on IPV perpetration was consistent even when controlling for changes in housing and relationship status and baseline age and reports of childhood exposure to abuse.

There is theoretical plausibility to the notion that food insecurity may lead to IPV perpetration. Stress theory suggests that a lack of material resources may lead to violence as stress depletes psychological resources required to enact self-control over the violence act.^11 35^ Stress theory has been supported in empirical studies from both low-income and high-income settings.^11 36–38^ In a broader ‘mental health’ lens that extends beyond only stress, we previously found food insecurity was related to greater depression symptoms, which in turn was associated with higher odds of IPV perpetration.^17^

Notwithstanding the longitudinal design of our study, it is plausible that other structural or historical factors drive both food insecurity and men’s use of IPV. We attempted to account for this by including fixed effects in the model, which control for within-person, unmeasured covariates. We also assessed results using clustering commands, which helps parse out distinctions between neighbourhoods. However, all of the neighbourhoods were within the peri-urban settlement, which has resource constraints that precludes generalising findings to other types of settings. A future study might examine other structural conditions that have been shown to predict both food insecurity and IPV, such as income inequality or racial discrimination.

Childhood abuse was strongly predictive of adult IPV perpetration. Child abuse is well-recognised as a major longitudinal driver of men’s later violence in adulthood.^39–41^ The reasons for this may be causal if those children who live in poorer households, with shifting household composition and childhood food insecurity, are at higher risk of childhood violence.^42–44^ They may also be associated if household conditions that are predictive of childhood abuse are also associated with adult poverty.

### Implications for programme and policy

These data suggest that household food insecurity may be a modifiable risk factor for men’s perpetrating IPV. Increasing access to food, through nutrition support, could attenuate men’s IPV perpetration, though experimental evidence for this is limited. One Mauritian study suggests that supplementing child nutrition can measurably reduce a father’s IPV perpetration,^45^ but it did not parse out the mechanisms through which nutrition might have led to this shift in violence.

Cash transfer programmes with women have shown that offering families a cash grant can reduce women’s IPV exposure,^46–48^ yet in a setting like South Africa where a large proportion of the population already depend on cash grants, it is unclear how additional transfers would alter rates of violence. Little research has explored food insecurity or economic interventions among men, but pilot studies suggest these programmes may reduce IPV perpetration.^49 50^ Despite potential downsides if economic interventions reinforce traditional male roles, concerns that an influx of cash might actually increase violence perpetration (by, eg, increasing alcohol intake) have not been borne out in the literature.^51^

One particularly interesting advance in development economics is the increased focus on assets and savings, rather than loans and credits. Increasing a family’s assets (through savings or individual development accounts, for example) seems to have a marked decrease in family stress.^52^ While savings approaches have begun to be tested among female survivors,^53 54^ our results suggest that they may be valuable for men. If coupled with gender transformative training, such savings and asset building programming could have health outcomes related to IPV perpetration.

### Limitations

Our data are drawn from a convenience sample of men recruited for a cluster randomised controlled trial, precluding the ability to generalise findings to the entire peri-urban settlement or other settings. The anonymity of ACASI may assist with accurate reporting of IPV by men by limiting social desirability bias. The data were collected as part of a larger cluster randomised trial, which we control for in analyses. One important assumption in fixed effects models is that the unmeasured confounders (denoted by) are accounted for so long as they stable over time.^55^ For example, study arm (intervention vs control) is stable over time and is thus incorporated in the estimate. However, it is possible that unmeasured time-variant confounders, such as crime experienced in daily life, do change over time.

Dynamic panel modelling is a within-person analysis approach that predicts how changes in one man’s food security status may relate to his perpetration of IPV over time. It does not, however, provide information about population-level considerations. Due to high levels of attrition over time there are limitations to the conclusions we can make, though the estimation strategy of maximum likelihood for missing values does partly address this concern. We cannot rule out bidirectionality fully, since it is possible that longer lags would have shown that changes in men’s violence use over time does influence their later food security status. However, within these timeframes, we can state the model fit for food insecurity driving later violence is stronger.

## Conclusion

We found that food insecurity is longitudinally associated with IPV perpetration among men. These findings can inform future violence prevention efforts, particularly in settings with high rates of endemic poverty. While a bidirectional relationship is plausible, our findings suggest that food insecurity seems to drive later violence rather than vice versa. Urgent policy and programmatic response to the intersecting issues of poverty and IPV can ensure health and well-being.

## Data Availability

Data are available in a public, open access repository. Data are available at : https://medat.samrc.ac.za/index.php/catalog/WW.
