# Supplementary material for: Food insecurity and men’s perpetration of partner violence in a longitudinal cohort in South Africa
Source: BMJ Nutr Prev Health. 2022 Feb 7;5(1):36–43. doi: 10.1136/bmjnph-2021-000288 (PMC9237862; doi:10.1136/bmjnph-2021-000288)
Supplement: Supplementary data [file bmjnph-2021-000288supp001.pdf]

Supplemental Table: Bidirectional cross-lagged dynamic panel model of food insecurity (n = 2,478)

	Coef	SE	p value
Time variant variables			
IPV perpetration intensity	0.02	0.02	0.276
Housing status	0.01	0.13	0.962
Time invariant variables			
Age at baseline	0.05	0.01	<0.001
Childhood abuse	0.05	0.01	<0.001
Alpha	2.00	22.20	0.928
**Fit indices**			
Chi2	5.13		
Chi2 p-value	0.40		
Degrees of freedom	4		
RMSEA	0.080		
CFI	0.661		
Coef: standardized coefficient; SE:standard error; IPV: intimate partner violence; RMSEA: root mean squared error of approximation; CFI: comparative fit index			
